# Can we unplug global health education from The Matrix?

**DOI:** 10.1371/journal.pgph.0004307

**Published:** 2025-02-25

**Authors:** Shashika Bandara, Ananya Tina Banerjee, Madhukar Pai

**Affiliations:** McGill School of Population and Global Health, McGill University, Montreal, Canada; PLOS: Public Library of Science, UNITED STATES OF AMERICA

In one of the most iconic scenes in the film The Matrix (2001), Neo, the protagonist is offered a choice between a red pill and a blue pill. This choice is offered by Morpheus, a leader of the resistance against the machines. In this story, machines have taken over the world. Human bodies are used as ‘batteries’ for electricity, and humans are kept alive by plugging them into a virtual world known as The Matrix. The blue pill allows Neo to stay within the illusion created by The Matrix, while the red pill would unplug him, show him the reality - and offer a choice to fight for liberation of humans.

The red pill and blue pill are metaphorical choices that represent a choice between critical examination of our status quo or remaining oblivious. The red pill has taken on various philosophical and social meanings since the release of The Matrix - and can be used as a tool in global health education to explain the two possible ways of teaching global health.

Within the last decade, a growing number of calls and acts of resistance have been made by the global health community, especially by students to change the curricula to reflect and address the deep power asymmetries in global health [1,2]. Essentially, these relentless calls ask us to ‘unplug’ global health education from ‘The Matrix,’ by providing an education that accurately represents the colonial histories and neo-colonial contemporary geopolitical and contextual factors affecting all determinants of health [2]. An argument can be made that the absence of such an education has directly or indirectly perpetuated downstream inequities we see today within representation, funding, global governance choices and more [3,4].

Building on these strong and necessary calls to action, we compare the usual ways of apolitical and ahistorical teaching that perpetuate the status quo (i.e., the blue pill way of teaching) versus the reimagined ways of teaching that consider the roots of global health inequities and challenges the status quo (i.e., the red pill way of teaching). While recognizing that global health curricula may not fall neatly into these binary categories, we hope that this juxtaposition can provoke a critical discussion we need to have to reconstruct global health education. We provide eight points of juxtaposition for consideration to educators (Table 1).

**Table 1:** Blue versus red pill versions of global health education.

Blue pill version of global health education – The Colonial Lens	Red pill version of global health education – The Decolonization Lens	Resources
Reinforces ‘White savior industrial complex’ mindset, where global health is seen as a charity exercise and providing aid to *save the poor and disadvantaged* without accountability or transparency	Combats the “white savior industrial complex,” where global health is understood as a collective goal of improving equity and dismantling structural discrimination via genuine partnerships, mutual trust and respect.	Banerjee et al., Lancet, 2023 Büyüm et al., BMJ GH, 2020 Bain et al., PLOS GPH Blog, 2024
Focuses mainly on technical and bio-medical issues about diseases, without connecting political, social, commercial and historical contexts	Actively includes the complex historical, geo-political, social and commercial aspects in contextualizing current challenges, solution building, policy making and delivery of care in global health.	Banerjee et al., Lancet, 2023 Bump & Aniebo, PLOS GPH, 2022 Kickbusch, BMJ, 2015
Emphasizes on the health and social inequities in low and middle income countries (LMICs), without much consideration of inequities within high income countries (HICs). Leads to showcasing LMICs in a deficit lens without highlighting strengths or expertise in LMICs.	Provides a deep understanding of inequities and injustices in all contexts among equity-denied communities that can be in parallel and cross-cutting. Discusses strengths and weaknesses of all systems including LMICs and HICs. Highlights best practices from LMICS and encourages bi-directional knowledge flow.	Anand & Pai, Lancet GH, 2023 Banerjee et al., Lancet, 2023
Minimizes discussion on the dynamics of power flow and the elite capture within the field of global health or the need to decolonize the field.	Engages with political determinants and unequal power distribution in global health. Pushes academic institutes to commit to decentralizing global health operations, by co-owning and transferring ownership to Global South institutes, when necessary.	Pai, Bandara & Kyobutungi, Lancet, 2024 Abimbola et al., PLOS Med, 2021 Sayegh et al., BMJ GH, 2022 Topp et al., BMJ GH, 2021 Kickbusch, BMJ, 2015
Dismisses power and privilege introspection, anti-racism and anti-oppression concepts and practices.	Engages critical consciousness of one’s own power and positionality to disrupt oppressive structures often grounded in colonialism and white supremacy.	Atkins et al. BMJ GH 2021 Krugman et al., BMJ GH 2022 Pai, Bandara & Kyobutungi, Lancet, 2024 Bandara & Banerjee, PLOS GPH, 2023
Offers student practicum or field work that is rooted in extractive practice, such as parachute research and short term medical missions.	Ensures sustainability and long-term partnerships with co-supervision of students from relevant country sites. Actively, aim to minimize harmful parachute research while promoting practices that benefits the local communities.	Gichane et al., Global Health Action, 2022 Astaidis, Yang et al. McGill Global Health Perspectives, 2021 Banerjee et al. Lancet 2023
Perpetuate epistemic injustice by ignoring or not paying attention to knowledge produced in the Global South and knowledge holders from the Global South, while favouring white, anglophone, Euro or Global North centric voices.	Prioritises the visibility of knowledge from the Global South and diverse ways of knowing. Also, centres the voices of experts in LMIC countries, racialized expertise Indigenous experts, youth and those from affected communities. Prioritises epistemic justice.	Banerjee et al., Lancet, 2023 Atkins et al., BMJ GH 2021 Bhakuni & Abimbola, Lancet GH, 2021 Pratt & de Vries, BMJ Journal of Medical Ethics, 2023 Krugman, PLOS GPH, 2023
Promotes the ‘HIC gaze’ to a student audience that is mostly Global North students, with voices of international students from the Global South or Indigenous students or racialized students often ignored.	Encourages students to unlearn and re-imagine global health that reflects cultural humility, allyship and solidarity.	Gichane et al., Global Health Action, 2022 Abimbola, BMJ GH, 2019 Abimbola, IRD Editions, 2024

First is tackling the perpetuation of “white saviour industrial complex” in the way global health is taught [5]. Coined by Teju Cole, this term indicates an ongoing feature of global health where participation is motivated by “big emotional experiences that validate privilege” with minimal consideration of justice [5]. There is a need to shift global health education away from the framing of global health and humanitarian aid as a global charity project built to save the poor, where the powerful and wealthy countries “help” the less powerful, on their own terms and benefit [2]. The current crisis in global health and development, caused by the Trump administration’s abrupt withdrawal from the World Health Organization, Paris Agreement and freezing of health aid, is a good example of why global health need this shift. The new framing posits global health should commit to a collective goal which aims to shift such structures of imbalance to address historical and contemporary injustices through reparations, genuine partnerships, power shifting and mutual trust building [2,5,6].

Second is confronting the sanitized version of global health where challenges are framed only as technical, medical or scientific problems without geopolitical or social contexts. The old way of framing largely focuses on disease burden or “silver bullet” solutions, often ignoring historical and contemporary contexts. The new framing intentionally and actively highlights geopolitical, historical, commercial and social contexts that tells the complete story behind the disease burden [5,7]. Examples of such teaching can include examining the genesis of tropical medicine as a colonial project or analyzing root causes of inequities that stem from a combination of historical injustices, structural, and systemic factors. A prime example is the global inequity in COVID-19 vaccine distribution during the pandemic.

Third is building an understanding that inequities exist everywhere and also exist transnationally - not just in Global South countries. Thus, global health courses need to counter the outdated deficit-centred framing of stark inequities existing only in low- and middle-income countries (LMICs) and reinforce the understanding that inequities are prevalent within high income countries (HICs) as well [5,8]. This approach allows us to address cross-cutting equity challenges better, learn from all countries, and help students view global health work as inclusive of addressing ‘glocal’ challenges [5,8]. This also creates further space for LMIC partners to share best practices and strengthens genuine global bi-directional knowledge flow.

Fourth is promoting the examination of power, including persuading institutions in the Global North to introspect on their role of perpetuating harmful power hierarchies [6,9,10]. The current status quo leans towards dismissing the need to examine power dynamics and encouraging the elite capture in global health [6,9,10]. Countering the process of dismissal via power analysis helps to highlight the value of shifting power towards the Global South [6].

A closely interconnected fifth challenge that deeply impacts global health institutions and instruction in the Global North is the trend of devaluing anti-racism, inclusion and other anti-oppression teachings and practices [6]. If we are to realistically push towards equitable global health structures, using a critical consciousness lens to consider our own power and positionalities to disrupt oppressive structures often grounded in white supremacy remain essential [1,11].

Another practice that needs to be reconsidered in global health education is the short-term global health missions, experiences that may be unsustainable and potentially harmful. As the sixth point we highlight the best practice of long-term partnerships with country partners in LMICs and equity-denied populations in HICs, geared towards benefiting local communities [12].

For global health curricula to be truly global, we must move away from seeing Euro-centric knowledge systems as the standard, where academics “defer to a distant, powerful, foreign gaze, whose power shapes our pose and what we can see or say” [13–15]. Thus, as our seventh point we highlight the need to center experts who are racialized, Indigenous, caste-oppressed from the Global South, and individuals in systemically marginalized communities in our courses [5,13]. We also need to share relevant knowledge that they have been producing as readings – shifting the knowledge monopoly away from the colonial knowledge structures [13].

Our final point builds on all the points above. For us to succeed, global health requires education curricula that actively encourages un-learning, uncomfortable dialogue, supports critical allyship, and cultural humility. This is the anti-thesis of an education system that minimizes voices of international or racialized or Indigenous students and promotes a singular high income country perspective of LMICs rooted in colonial and neo-colonial ethos.

In summary, in this commentary, we compare the “red pill” versus “blue pill” versions of global health education which represents the choice to radically reimagine global health education or remain comfortable with the status quo, respectively. We invite educators, including us, to take the “red pill”, to critically examine our global health curricula and find ways to reimagine them. We note that shifting global health education is not as easy as taking the “red pill.” To truly imagine a red pill version (a more just, inclusive, collaborative form) of global health education, we need a collective, continuous, and long-term effort.
